# Micro-costing analysis of guideline-based treatment by direct-acting agents: the real-life case of hepatitis C management in Brazil

**DOI:** 10.1186/s12876-017-0676-8

**Published:** 2017-11-23

**Authors:** Hugo Perazzo, Marcelino Jose Jorge, Julio Castro Silva, Alexandre Monken Avellar, Patrícia Santos Silva, Carmen Romero, Valdilea Gonçalves Veloso, Ruben Mujica-Mota, Rob Anderson, Chris Hyde, Rodolfo Castro

**Affiliations:** 1 Fundação Oswaldo Cruz, FIOCRUZ, Instituto Nacional de Infectologia Evandro Chagas, INI, Laboratório de Pesquisa Clínica em DST e AIDS, LAPCLIN-AIDS, Rio de Janeiro, Brazil; 2 Fundação Oswaldo Cruz, FIOCRUZ, Instituto Nacional de Infectologia Evandro Chagas, INI, Laboratório de Pesquisa em Economia das Organizações de Saúde, LAPECOS, Rio de Janeiro, Brazil; 3 Fundação Oswaldo Cruz, FIOCRUZ, Instituto Nacional de Infectologia Evandro Chagas, INI, Plataforma de Pesquisa Clínica, Rio de Janeiro, Brazil; 4 Fundação Oswaldo Cruz, FIOCRUZ, Centro de Desenvolvimento Tecnológico em Saúde, CDTS, Rio de Janeiro, Brazil; 5 University of Exeter Medical School, UEMS, Evidence Synthesis & Modelling for Health Improvement, ESMI, Exeter, UK; 6 Universidade Federal do Estado do Rio de Janeiro, UNIRIO, Instituto de Saúde Coletiva, ISC, Rio de Janeiro, Brazil

**Keywords:** Chronic hepatitis C, Treatment, Micro-costing

## Abstract

**Background:**

Eradication of hepatitis C virus (HCV) using direct-acting agents (DAA) has been associated with a financial burden to health authorities worldwide. We aimed to evaluate the guideline-based treatment costs by DAAs from the perspective of the Brazilian Ministry of Health (BMoH).

**Methods:**

The activity based costing method was used to estimate the cost for monitoring/treatment of genotype-1 (GT1) HCV patients by the following strategies: peg-interferon (PEG-IFN)/ribavirin (RBV) for 48 weeks, PEG-IFN/RBV plus boceprevir (BOC) or telaprevir (TEL) for 48 weeks, and sofosbuvir (SOF) plus daclastavir (DCV) or simeprevir (SIM) for 12 weeks. Costs were reported in United States Dollars without (US$) and with adjustment for purchasing power parity (PPP$). Drug costs were collected at the National Database of Health Prices and an overview of the literature was performed to assess effectiveness of SOF/DCV and SOF/SIM regimens in real-world cohorts.

**Results:**

Treatment costs of GT1-HCV patients were PPP$ 43,176.28 (US$ 24,020.16) for PEG-IFN/RBV, PPP$ 71,196.03 (US$ 39,578.23) for PEG-IFN/RBV/BOC and PPP$ 86,250.33 (US$ 47,946.92) for PEG-IFN/RBV/TEL. Treatment by all-oral interferon-free regimens were the less expensive approach: PPP$ 19,761.72 (US$ 10,985.90) for SOF/DCV and PPP$ 21,590.91 (US$ 12,002.75) for SOF/SIM. The overview reported HCV eradication in up to 98% for SOF/DCV and 96% for SOF/SIM.

**Conclusion:**

Strategies with all oral interferon-free might lead to lower costs for management of GT1-HCV patients compared to IFN-based regimens in Brazil. This occurred mainly because of high discounts over international DAA prices due to negotiation between BMoH and pharmaceutical industries.

**Electronic supplementary material:**

The online version of this article (10.1186/s12876-017-0676-8) contains supplementary material, which is available to authorized users.

## Background

Chronic hepatitis C (CHC) remains a major public health issue that might lead to cirrhosis and its complications, such as portal hypertension and hepatocellular carcinoma [1]. Eradication of hepatitis C virus (HCV) has been associated with lower rates of liver-related complications [2]. Transient elastography (TE) by FibroScan® (EchoSens, Paris, France) is a validated method to liver fibrosis assessment [3] and seems to be cost-effective to diagnose cirrhosis compared to liver biopsy [4]. The high efficacy of direct-acting antiviral drugs (DAAs) has revolutionized the management of patients with CHC. High rates of sustained virological response (SVR), defined as undetectable serum HCV-RNA levels 12 weeks after completing treatment, has been obtained by DAA treatment in patients with advanced liver fibrosis or compensate cirrhosis [5]. However, the decrease of the burden of liver disease in CHC by DAA treatment might be associated with high costs to health authorities worldwide [6]. Public health strategies should be implemented to promote primary care access to interferon-free regimes, especially in low to middle-income countries with high prevalence of CHC.

Drug prices are very important in HCV management but they are clearly not the only costs to be considered in the analysis [7]. The decision to incorporate new drugs in guidelines for CHC management should take into account the cost and cost-effectiveness analysis. The evidence base from economic evaluations of new healthcare technologies has been limited by the quality of the available data, strengths and weaknesses of different methods used for cost determination and differences among public health systems worldwide [8]. In Brazil, it is estimated that 1.4 to 1.7 million of people are living with CHC [9], most of them genotype-1 (GT1) HCV [10] and up to 20% with advanced fibrosis or cirrhosis [11]. Recently, the Brazilian Ministry of Health (BMoH) has recommended the use of DAAs for CHC treatment due to high discounts given by pharmaceutical companies. Since July 2015, patients with advanced fibrosis (METAVIR F ≥ 3) have free access to these drugs [12]. However, few studies have investigated the cost for CHC treatment with DAAs in a low to middle-income country. The aim of this study was to evaluate the guideline-based treatment costs by DAAs from the perspective of a public national reference healthcare institute in Brazil. In addition, we reviewed the literature for effectiveness of all-oral interferon-free regimens available in Brazil in real-life cohort studies.

## Methods

### Study design and setting

This cost-analysis study used cost data collected in a tertiary center for treatment of infectious diseases: National Institute of Infectious Diseases Evandro Chagas-Oswaldo Cruz Foundation (INI/FIOCRUZ) at Rio de Janeiro, Brazil. INI/FIOCRUZ has been playing an important role for the BMoH in development of public health policies and strategies for prevention and treatment of infectious diseases, including viral hepatitis. In the Brazilian Public Health System, known as *Sistema Único de Saúde* (SUS), individuals can have free access to HCV treatment whether confirmed presence of liver fibrosis. According to the recommendations for HCV treatment from the BMoH, treatment was delivery for free in patients with liver fibrosis stages F ≥ 2 until 2012. However, from 2013 to nowadays, the threshold for free-treatment access has changed for fibrosis stages F ≥ 3. This analysis simulated follow-up and antiviral regimens exclusively for treatment of GT1-HCV patients according to the recommendation of BMoH from 2011 to 2015.

### Method for cost determination

The activity based costing (ABC) method was used for cost determination. This micro-costing method identifies different activities that the institution performs and includes the corresponding indirect costs (e.g. management costs) to estimate total cost of a procedure. ABC method recognizes the relationship between costs, activities (called cost drivers) and products (exams or medical visit) taking into account this relationship in the costing analysis [13]. Secondary data were obtained from: (i) public access production resources from the Department of Statistics; (ii) inventory of permanent material; (iii) records of expenditures with resources from the Financial Department for a one-year (2013) period (to correct the potential seasonal expenses due to diseases outbreaks in infectious diseases); and (iv) cost of 450 diagnostic tests and 100 sub-types of clinical procedures. Administrative costs were estimated through the determination of the proportional contribution of each activity to the pooled expenses and considered for all diagnostic tests and clinical procedures. Prices in national currency [Brazilian Real (BRL)] and inflated to average prices of July of 2015 in United States Dollars according to the World Bank, without (exchange rate = 3.11) and with adjustment for purchasing power parity (PPP adjustment rate = 1.73). We report these as the “nominal price” (US$) and the “PPP-adjusted price” (PPP$), respectively.

### Costs of personnel, blood analysis tests and medical visits

Personnel costs for each procedure were calculated based on the annual salary payment of each employee and the working time spent in the specific activity. At INI/FIOCRUZ, salaries are determined according to employee’s specialization (i.e., people with PhD degree have higher salaries compared to those without) for individuals working in similar activities. Regarding expenditures with blood analysis tests, we considered costs of materials needed to perform the test, such as kits and reagents. Methodology for cost determination of non-invasive methods for liver fibrosis assessment were detailed in the Supplementary Material. Briefly, the following aspects were taken into account to estimate the cost per unit of TE (i) the price and depreciation of the FibroScan® machine; (ii) annual maintenance by M probe calibration; (iii) salaries of personnel who perform the examination and (iv) indirect administrative costs. We reported the price of TE adjusted with the opportunity cost of using public resources for implementation of new technologies (cost of capital). Costs with specific material for liver biopsy [Menghini needle 16G (Biomedical, Florence, Italy)] and histological analysis, as well as personnel costs and cost for a day-clinic hospitalization were considered for estimation of total cost to perform a liver biopsy. The market prices were considered for diagnostic tests not performed at INI/FIOCRUZ.

### Cost of antiviral drugs for CHC treatment

Drug costs were collected in a web-based public domain site that describes the prices paid by the BMoH for all drugs incorporated in the Public Health System and purchased from the pharmaceutical industry (Database of Health Prices – “*Banco de Preços em Saúde*” - accessible at: http://www.saude.gov.br/bps). A weighted-mean price for each antiviral drug was estimated taking into account all drug payments made by the BMoH from January to December 2015. We then calculated the price for a single-dose of PEG-interferon α-2a 180 mcg (PEG-IFN) [Pegasys® - Roche] and a single capsule or tablet for ribavirin (RBV) [generic by BMoH], boceprevir (BOC) [Victrelis® – Merk], telaprevir (TEL) [Incivek® - Vertex Pharmaceuticals], sofosbuvir (SOF) [Sovaldi® – Gilead], daclastavir (DCV) [Daklinza® - Bristol-Myers Squibb] and simeprevir (SIM) Olysio® – Janssen Pharmaceuticals].

### Strategies for treatment of GT1-HCV patients according to the BMoH guidelines

In the present study, we compared the strategies for HCV treatment of GT1 patients as recommended by the BMoH from 2011 to 2015. During this period, Brazilian guidelines for CHC treatment were published in 2011, 2013 and 2015. In 2011, GT1 patients should be treated by PEG-IFN plus RBV for 48 weeks and a liver biopsy with significant fibrosis (METAVIR F ≥ 2) was necessary for treatment access (regimen BMoH-2011) [14]. In 2013, presence of advanced fibrosis (liver biopsy METAVIR F ≥ 3 or TE ≥ 9.6 kPa) allowed the addition of first generation protease inhibitors [BOC for 44 weeks (regimen BMoH-2013A) or TEL for 12 weeks (regimen BMoH-2013B)] to PEG-IFN/RBV during 48 weeks [15]. More recently, in 2015, all-oral and interferon-free regimens [SOF plus DCV for 12 weeks (regimen BMoH-2015A) or SOF plus SIM for 12 weeks (regimen BMoH-2015B)] were recommended for GT1 HCV patients. Patients with decompensated cirrhosis, HIV co-infection or previous failure with PEG-IFN/RBV ± BOC or TEL might be treated by SOF/DCV 24 weeks. In addition, serological biomarkers or TE could replace liver biopsy for free-access to DAA treatment [12]. In all scenarios, liver fibrosis was staged for treatment access and patients were followed with blood tests and medical visits. Table 1 summarizes the strategies for treatment of GT1 HCV patients according to the BMoH guidelines from 2011 to 2015.

**Table 1:** Description of strategies for treatment of genotype 1 chronic hepatitis C according to the Brazilian Ministry of Health from 2011 to 2015

Guideline	Fibrosis staging	Prerequisite for treatment	Drugs	Duration (weeks)	Doses	Interventions
BMoH-2011	Liver biopsy	METAVIR F ≥ 2	PEG-IFN α-2a 180 mcg [Pegasys® - Roche]	48	1 injection weekly	PEG-RBV for 48 weeks. Medical visit at screening, baseline, W2, W4 and every 4 weeks for W4 to W48. Response-guided therapy or stopping rules based on HCV-RNA at W4, W12 and W24. SVR at W72
			RBV 250 mg [BMoH generic]	48	5 capsules daily	
BMoH-2013A	TE	LSM ≥ 9.6 kPa	PEG-IFN α-2a 180 mcg [Pegasys® - Roche]	48	1 injection weekly	Leading-phase with PEG-RBV for 4 weeks and PEG-RBV/BOC for 44 weeks. Medical visit at screening, baseline, W2, W4, W5, W6 and W8, and every 4 weeks from W8 to W48. Response-guided therapy or stopping rules based on HCV-RNA on W4, W12 and W24. SVR at W72
			RBV 250 mg [BMoHgeneric]	48	5 capsules daily	
			BOC 200 mg [Victrelis® – Merk]	44	12 tablets daily	
BMoH-2013B	TE	LSM ≥ 9.6 kPa	PEG-IFN α-2a 180 mcg [Pegasys® - Roche]	48	1 injection weekly	PEG-RBV/TEL for 12 weeks and PEG-RBV for 36 weeks. Medical visit at at screening, baseline, W1, W2 and W4, then every 4 weeks from W4 to W48. Response-guided therapy or stopping rules based on HCV-RNA on W4, W12 and W24. SVR at W72
			RBV 250 mg [BMoH generic]	48	5 capsules daily	
			TEL 375 mg [Incivek ® - Vertex]	12	6 tablets daily	
BMoH-2015A	TE or APRI or FIB-4	LSM ≥ 9.6 kPa or APRI ≥1.5 or FIB-4 ≥ 3.25	SOF 400 mg [Sovaldi® – Gilead]	12	1 tablet daily	SOF/DCV for 12 weeks. Medical visits at screening, baseline, W2, W4, W8 and W12. No response-guided therapy or stopping rules. SVR at W24
			DCV 60 mg [Daklinza® - BMS]	12	1 tablet daily	
BMoH-2015B	TE or APRI or FIB-4	LSM ≥ 9.6 kPa or APRI ≥1.5 or FIB-4 ≥ 3.25	SOF 400 mg [Sovaldi® – Gilead]	12	1 tablet daily	SOF/SIM for 12 weeks or 24 weeks in presence of decompensated cirrhosis (Child-Pugh B/C). Medical visits at screening, baseline, W2, W4, W8 and W12. No response-guided therapy or stopping rules. SVR at W24
			SIM 150 mg [Olysio – Janssen®]	12	1 tablet daily	

*APRI* aspartate-to-platelet index, *BMoH* Brazilian Ministry of Health, *BOC* boceprevir, *DCV* daclastavir, *FIB-4* fibrosis-4 score, *kPa* kilopascal, *LSM* liver stiffness measurement, *PEG-IFN* peginterferon, *RBV* ribavirin, *SIM* simeprevir, *SOF* sofosbuvir, *SVR* sustained-virologic response, *TE* transient elastography, *TEL* telaprevir

### Literature review for efficacy of SOF/DCV and SOF/SIM regimens in real-life scenario

We systematically searched the references using the following predetermined inclusion criteria: cohort study that included GT1 HCV patients treated in real-life scenario by SOF/DCV or SOF/SIM ± RBV for 12 weeks. This search was performed in June 2017 at MEDLINE (PubMed) (1966–2017) using the following terms: [“sofosbuvir” OR “Sovaldi®” OR “GS-7977” OR “simeprevir” OR “Olysio®” OR“TMC-435” OR “daclastavir” OR “Daklinza®” OR “BMS-790052”] AND [“hepatitis C”] AND [“real-world” OR “real-life”] (Additional file 1: Table S1).

## Results

### Costs for liver fibrosis assessment

The estimated costs of TE with personnel, machine and administrative costs were PPP$ 40.08, 29.80 and 24.78, respectively. The nominal and PPP-adjusted costs of a single TE exam were PPP$ 95.32 (US$ 53.32). The adjustment using the opportunity cost lead to an increase in up 50% of TE price (Additional file 1: Table S2). Costs of APRI and FIB-4 were estimated at PPP$ 27.85 (US$ 15.48) and PPP$ 36.58 (US$ 20.33), respectively. Total cost for liver biopsy was PPP$ 834.41 (US$ 482.32) including material to perform liver biopsy and histological analysis [PPP$ 80.75 (US$ 46.68)], personnel costs [PPP$ 301.47 (US$ 174.26)] and cost for day-clinic hospitalization [PPP$ 452.19 (US$ 261.38)].

### Costs for GT1 HCV treatment

The estimated price of GT1 HCV treatment drugs was an important source of variability in the total cost for HCV treatment. PEG-IFN α-2a 180 mcg cost PPP$ 648.00 (US$ 360.22) per weekly injection and RBV was the least expensive drug costing PPP$ 106.05 (US$ 58.80) per week. The regimen PEG-IFN (180 mcg SC weekly)/RBV (1250 mg PO daily if body weight ≥ 75 kg) cost PPP$ 754.05 or US$ 419.18 per week. Considering the first generation protease inhibitors (PI), TEL had a higher cost compared to BOC [PPP$ 87.01 (US$ 48.37) vs PPP$ 7.96 (US$ 4.42) per tablet). The weekly costs for treatment by BOC (800 mg PO q8hr) and TEL (750 mg PO q8hr) were PPP$ 668.64 and 3654.42, respectively. Thus, the weekly costs for PEG-IFN/first generation PI regimens ranges from PPP$ 1422.69 (with BOC) and 4408.47 (with TEL). Considering the DAAs, the NS5A inhibitor DCV 60 mg and the second generation PI SIM 150 mg cost PPP$ 388.78 (US$ 206.09) and PPP$ 541.24 (US$ 300.86) per week, respectively. In addition, SOF 400 mg, a NS5B polymerase inhibitor, had a PPP-adjusted and a nominal cost of PPP$ 1014.23 and US$ 563.78 weekly, respectively. The regimens SOF (400 mg PO daily)/DCV (60 mg PO daily) and SOF (400 mg PO daily)/SIM (150 mg PO daily) cost PPP$ 1403.01 and US$ 1555.47 per week, respectively. Table 2 summarizes the nominal and PPP-adjusted costs per unit for tests, medical care and drugs used in GT1 HCV patient management.

**Table 2:** Cost per unit and number of procedures for monitoring and injections/tablets for treatment of genotype 1 chronic hepatitis C according to different regimens recommended by the BMoH from 2011 to 2015

	Cost per unit		PEG-RBV	PEG-RBV/BOC	PEG-RBV/TEL	SOF/DCV	SOF/SIM
	US$	PPP$	n	n	n	n	n
Transient elastography	53.35	95.32	–	1	1	1	1
Liver biopsy	482.32	834.41	1	–	–	–	–
Blood analysis tests							
WBC. RBC and platelet	10.62	19.11	15	17	16	6	6
Fasting glucose	4.86	8.75	6	6	6	6	6
BUN/creatinine	4.84	8.70	15	16	15	6	6
ALT	4.85	8.73	15	16	15	6	6
AST	4.86	8.74	15	16	15	6	6
Alkaline phosphatases	4.77	8.58	6	6	6	6	6
GGT	4.83	8.69	6	6	6	6	6
Total bilirubin	4.84	8.71	6	6	6	6	6
Albumin	5.07	9.12	6	6	6	6	6
INR	6.68	12.02	6	13	14	6	6
HCV genotype	156.33	281.22	1	1	1	1	1
HCV-RNA	87.05	156.60	8	7	7	2	2
TSH	26.26	47.23	6	6	6	–	–
Beta-hCG	45.52	81.88	4	4	4	4	4
Medical follow-up							
GP’s medical visit	54.54	98.11	1	1	1	1	1
Specialist’s medical visit	62.92	113.19	14	14	14	5	5
Blood sample collection	37.83	68.06	15	15	15	6	6
Nursing consultation	31.83	57.26	5	5	5	4	4
Drugs							
PEG-IFN α-2a 180 mcg – SC injection	360.22	648.00	48	48	48	–	–
Ribavirin 250 mg – PO capsule	1.68	3.03	1680	1680	1680	–	–
Boceprevir 200 mg – PO tablet	4.42	7.96	–	3612	–	–	–
Telaprevir 375 mg – PO tablet	48.37	87.01	–	–	504	–	–
Sofosbuvir 400 mg – PO tablet	80.54	144.89	–	–	–	84	84
Daclastavir 60 mg – PO tablet	30.87	55.54	–	–	–	84	–
Simeprevir 150 mg - PO tablet	42.98	77.32	–	–	–	–	84

Costs in United States Dollars (US$) and with purchasing power parity (PPP$) on July 2015. Drug costs estimated as the weighted-mean price (http://www.saude.gov.br/bps)

*BOC* boceprevir, *DCV* daclastavir, *PEG-IFN* peginterferon, *RBV* ribavirin, *SIM* simeprevir, *SOF* sofosbuvir, *TEL* telaprevir, *WBC* white blood cell, *RBC* red blood cell, *BUN* blood urea nitrogen, *ALT* alanine aminotransferase, *AST* aspartate aminotransferase, *AP* alkaline phosphatases, *GGT* gamma-glutamyl transpeptidase, *INR* international normalized ratio, *TSH* thyroid-stimulating hormone, *hCG* human chorionic gonadotropin, *GP* general practicioner

Treatment regimens according to BMoH guidelines: PEG-RBV 48w [BMoH-2011]; PEG-RBV (48w)/BOC 44w [BMoH-2013A]; PEG-RBV (48w)/TEL (12w) [BMoH-2013B]; SOF/DCV 12w [BMoH-2015A] and SOF/SIM 12w [BMoH-2015B]

According to the procedures recommended at the BMoH’s guidelines, treatment with PEG-IFN/RBV/first generation PI [BMoH-2013A and B] was the most expensive approach. In those recommendations, patients were treated for 48 weeks by PEG-IFN/RBV and BOC for 44 weeks [total of PPP$ 71,196.03 (US$ 39,578.23) per patient] or TEL for 12 weeks [total of PPP$ 86,250.33 (US$ 47,946.92) per patient]. Treatments by new all-oral interferon-free regimens were the least expensive approach: the costs for SOF/DCV [BMoH-2015A] and SOF/SIM [BMoH-2015B] were PPP$ 19,761.72 (US$ 10,985.90) and PPP$ 21,590.91 (US$ 12,002.75), respectively (Table 3). In the presence of decompensated cirrhosis (Child-Pugh B/C), the necessity to treat with SOF/DCV for 24 weeks leads to an 84% increase in total costs [PPP$ 36,597.71 (US$ 20,345.04)]. In all scenarios, the drug costs were responsible by approximatively 90% of total cost for treat patients with CHC. The cost with medical visits and blood analysis tests fell by up to 40% in treatment with DAAs [guidelines BoMH-2015A/B] compared to regimens that used PEG-IFN [guidelines BMoH 2011 and 2013A/B].

**Table 3:** Monitoring and drugs costs (US$ and PPP-adjusted US$) for treatment of genotype-1 patient with chronic hepatitis C according to different regimens recommended by the BMoH from 2011 to 2015

	PEG-RBV		PEG-RBV/BOC		PEG-RBV/TEL		SOF/DCV		SOF/SIM	
	US$	PPP$	US$	PPP$	US$	PPP$	US$	PPP$	US$	PPP$
TE	–	–	53.35	95.32	53.35	95.32	53.35	95.32	53.35	95.32
Liver biopsy	482.32	834.41	–	–	–	–	–	–	–	–
Total	482.32	834.41	53.35	95.32	53.35	95.32	53.35	95.32	53.35	95.32
Blood analysis tests										
WBC. RBC and platelet	159.39	286.72	180.64	324.95	170.02	305.84	63.76	114.69	63.76	114.69
Fasting glucose	29.20	52.52	29.20	52.52	29.20	52.52	29.20	52.52	29.20	52.52
BUN/creatinine	72.58	130.56	77.41	139.26	72.58	130.56	29.03	52.22	29.03	52.22
ALT	72.78	130.92	77.63	139.65	72.78	130.92	29.11	52.37	29.11	52.37
AST	72.91	131.16	77.78	139.91	72.91	131.16	29.17	52.47	29.17	52.47
AP	28.61	51.46	28.61	51.46	28.61	51.46	28.61	51.46	28.61	51.46
GGT	28.99	52.15	28.99	52.15	28.99	52.15	28.99	52.15	28.99	52.15
Total bilirubin	29.04	52.24	29.04	52.24	29.04	52.24	29.04	52.24	29.04	52.24
Albumin	30.44	54.75	30.44	54.75	30.44	54.75	30.44	54.75	30.44	54.75
INR	40.10	72.14	86.89	156.30	93.57	168.32	40.10	72.14	40.10	72.14
HCV genotype	156.33	281.22	156.33	281.22	156.33	281.22	156.33	281.22	156.33	281.22
HCV-RNA	696.43	1252.80	609.38	1096.20	609.38	1096.20	174.11	313.20	174.11	313.20
TSH	157.55	283.41	157.55	283.41	157.55	283.41		–		–
Beta-hCG	182.07	327.53	182.07	327.53	182.07	327.53	182.07	327.53	182.07	327.53
Total	1756.41	3159.58	1751.94	3151.54	1733.45	3118.27	849.94	1528.95	849.94	1528.95
Medical follow-up										
GP’s medical visit	54.54	98.11	54.54	98.11	54.54	98.11	54.54	98.11	54.54	98.11
Specialist’s visit	880.95	1584.73	880.95	1584.73	880.95	1584.73	314.62	565.97	314.62	565.97
Blood sample	567.52	1020.90	567.52	1020.90	567.52	1020.90	227.01	408.36	227.01	408.36
Nursing consultation	159.15	286.29	159.15	286.29	159.15	286.29	127.32	229.03	127.32	229.03
Total	1662.15	2990.02	1662.15	2990.02	1662.15	2990.02	723.49	1301.47	723.49	1301.47
Drugs										
PEG-IFN 180 mcg	17,290.59	31,103.78	17,290.59	31,103.78	17,290.59	31,103.78	–	–	–	–
Ribavirin 250 mg	2828.69	5088.49	2828.69	5088.49	2828.69	5088.49	–	–	–	–
Boceprevir 200 mg	–	–	15,991.51	28,766.88	–	–	–	–	–	–
Telaprevir 375 mg	–	–	–	–	24,378.69	43,854.45	–	–	–	–
Sofosbuvir 400 mg	–	–	–	–	–	–	6765.68	12,170.68	6765.68	12,170.68
Daclastavir 60 mg	–	–	–	–	–	–	2593.44	4665.30	–	–
Simeprevir 150 mg	–	–	–	–	–	–	–	–	3610.29	6494.49
Total	20,119.28	36,192.27	36,110.79	64,959.15	44,497.97	80,046.72	9359.12	16,835.98	10,375.97	18,665.17
TOTAL COST	24,020.16	43,176.28	39,578.23	71,196.03	47,946.92	86,250.33	10,985.90	19,761.72	12,002.75	21,590.91

Costs in United States Dollars (US$) and with purchasing power parity (PPP$) on July 2015. Drug costs estimated as the weighted-mean price (http://www.saude.gov.br/bps)

*PEG-IFN* peginterferon, *WBC* white blood cell, *RBC* red blood cell, *BUN* blood urea nitrogen, *ALT* alanine aminotransferase, *AST* aspartate aminotransferase, *AP* alkaline phosphatases, *GGT* gamma-glutamyl transpeptidase, *INR* international normalized ratio, *TSH* thyroid-stimulating hormone, *hCG* human chorionic gonadotropin, *GP* general practicioner

Treatment regimens according to BMoH guidelines: PEG-RBV 48w [BMoH-2011]; PEG-RBV (48w)/BOC 44w [BMoH-2013A]; PEG-RBV (48w)/TEL (12w) [BMoH-2013B]; SOF/DCV 12w [BMoH-2015A] and SOF/SIM 12w [BMoH-2015B]

### Literature overview for efficacy of SOF/DCV and SOF/SIM regimens

**Fig. 1:**
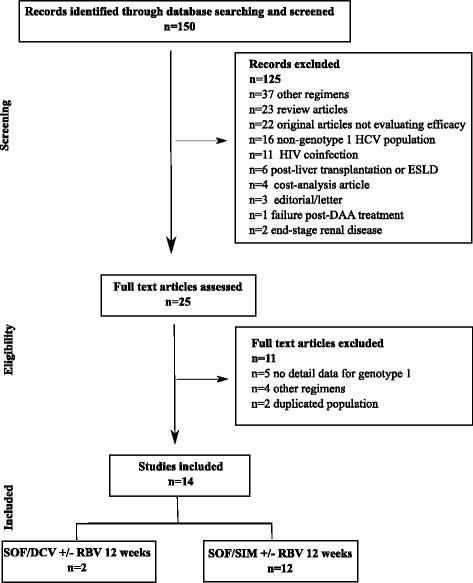
Flow-chart of study selection for the literature overview

Out of 150 identified articles, 25 references were eligible and 14 real-life cohort studies that reported the efficacy of SOF/DCV (*n* = 2) or SOF/SIM ± RBV (*n* = 12) in GT1 HCV patients were included (Fig. 1). Overall, SVR rates for SOF/DCV ± RBV and SOF/SIM ± RBV during 12 weeks ranged from 83 to 98% [16, 17] and from 75 to 96% [18–29], respectively. SVR rates were lower in presence of cirrhosis compared to non-cirrhotic patients (87% vs 98% with SOF/DCV [17] and 81% vs 91% with SOF/SIM [26]. Higher SVR rates can be achieved in cirrhotic patients when extending SOF/DCV for 24 weeks [17] or adding RBV to SIM/SOF [24]. Pol et al. reported lower SVR rates in GT1a patients compared to GT1b (89% vs 95% with SOF/DCV 12 weeks) that might be not significant when extending treatment for 24 weeks (95% for both) [17]. Similar results were observed in patients treated by SOF/SIM 12 weeks (83% for GT1a vs 90% for GT1b) [26]. Table 4 summarizes SVR rates from real-life cohort studies that analysed the efficacy of SOF/DCV and SOF/SIM regimens in GT1 patients stratified by GT1 subtype and presence or absence of cirrhosis or previous treatment.

**Table 4:** Real-life cohort studies that evaluated efficacy (SVR12) of Sofosbuvir/Daclastavir (SOF/DCV) or Sofosbuvir/Simeprevir (SOF/SIM) regimens for treatment of HCV mono-infected genotype-1 patients

Authors	Year	Local	Regimen	Overall		Non-cirrhosis		Cirrhosis		Naive		Experimented		GT 1a		GT 1b	
				n	SVR	n	SVR	n	SVR	n	SVR	n	SVR	n	SVR	n	SVR
Sofosbuvir (400 mg)/Daclastavir (60 mg) regimen studies																	
Pol et al. [17]	2017	France	SOF/DCV 12w	160	92%	66	98%	94	87%	64	88%	96	95%	73	95%	83	89%
			SOF/DCV 24w	439	95%	97	97%	342	94%	48	85%	391	96%	210	95%	209	95%
Welzel et al. [16]	2016	Europe	SOF/DCV ± RBV 12w	319	98%	–	–	–	–	–	–	–	–	151	99%	155	97%
Sofosbuvir (400 mg)/Simeprevir (150 mg) regimen studies																	
Marino et al. [[28]	2017	Spain	SOF/SIM ± RBV 12w	835	92%	–	–	835	92%	–	–	–	–	–	–	–	–
Ramos et al. [29]	2017	Spain	SOF/SIM 12w	179	93%	–	–	–	–	–	–	–	–	–	–	–	–
Chang et al. [27]	2017	USA	SOF/SIM 12w	21	91%	–	–	–	–	–	–	–	–	–	–	–	–
Sulkowski et al. [26]	2016	USA	SOF/SIM 12w	639	85%	272	91%	367	81%	65	–	–	–	371	83%	185	90%
Lutchtman et al. [25]	2016	USA	SOF/SIM 12w	148	82%	43	88%	193	78%	62	87%	86	78%	94	79%	42	93%
Pellicelli et al. [24]	2016	Italy	SOF/SIM/RBV 12w	270	96%	–	–	270	96%	–	–	–	–	–	–	–	–
Roytman et al. [23]	2016	USA	SOF/SIM 12w	138	89%	43	93%	95	87%	76	87%	55	92%	–	–	–	–
Thornton et al. [22]	2016	USA	SOF/SIM 12w	114	89%	–	–	–	–	–	–	–	–	–	–	–	–
Jayasekera et al. [19]	2016	USA	SOF/SIM 12w	35	86%	13	92%	22	82%	7	86%	28	86%	–	–	–	–
Pillail et al. [20]	2016	USA	SOF/SIM ± RBV 12w	113	84%	23	91%	90	82%	57	89%	56	79%	–	–	–	–
Barron et al. [21]	2016	USA	SOF/SIM ± RBV 12w	35	83%	–	–	–	–	–	–	–	–	–	–	–	–
Backus et al. [18]	2015	USA	SOF/SIM 12w	1130	75%	664	81%	462	68%	699	78%	431	71%	632	73%	366	80%

*DCV* daclastavir, *GT* genotype, *SIM* simeprevir, *SOF* sofosbuvir, *SVR* sustained virological response, *w* week

## Discussion

The present study highlighted that GT1 HCV treatment by effective DAAs might be less expensive than interferon-based regimens in a low-to-middle income country, mainly due to price negotiation between health authorities and pharmaceutical companies and lower costs with monitoring in the all-oral interferon-free treatments.

The cost of TE was increased due to the very high interest rates used in Brazil [30, 31] and higher expenses with personnel at INI/FIOCRUZ. TE could be less costly in countries with lower opportunity cost of public funds and in those where nurses perform the exams. Liver fibrosis staging by TE seems to be cost-effective compared to liver biopsy and it was priced from US$ 21 to US$ 73 in the United Kingdom and up to US$ 145 in the United States [4].

HCV eradication has been associated with a financial burden, especially due to high DAAs costs which are extremely variable worldwide [6]. An analysis of for a 12-week SOF course price (PPP-adjusted) in 30 countries described that Poland (PPP$ 101,063.00), Turkey (PPP$ 91,339) and United States (PPP$ 64,680) were the top three ranking countries with the most expensive prices. On the other hand, India (PPP$ 1861), Mongolia (PPP$ 2604) and Egypt (PPP$ 3117) were the countries with the least expensive price for SOF-therapy [32]. In 36 clinical sites in USA, real-world costs for PEG-IFN/RBV/BOC 48 weeks, PEG-IFN/TEL 48 weeks and SOF/SIM 12 weeks were US$ 57,960; US$ 113,400; US$ 180,600; respectively. In addition, the mean cost per SVR in all-oral interferon-free was higher in patients with advanced fibrosis (US$ 167,467 for F3F4 vs 116,579 for F0F1F2) [33]. However, both studies only included cost of HCV medications, not adjunctive medications, hospital costs, or projected medical costs. Data from Europe and Canada reported a total cost [mean (95%CI)] of €14,559 (13,323–15,836) per patient and €38,514 (35,244–41,892) per SVR for IFN-based regimens considering treatment, monitoring and complications [34]. The price disparities among countries might be explained partly by the pharmaceutical price-setting policies used in different countries and negotiation between health authorities and pharmaceutical companies. Some countries set prices according to explicit cost-effectiveness thresholds based on gross domestic product (GDP) per capita resulting in high prices without consideration of budget impact [35]. Therefore, DAA treatment costs are lower in Brazil compared to others countries mainly due to intense negotiation of BMoH with pharmaceutical companies resulting in more than 90% discount over international prices for SOF, DCV and SIM [9].

In the present study, the reduced price for DAAs and shorten treatment duration might explain the lower total cost for HCV eradication with all-oral interferon-free compared to IFN-based regimens. Weekly prices for PEG-IFN (US$ 360 or PPP$ 648), BOC (US$ 371 or PPP$ 672) and TEL (US$ 2030 or PPP$ 3654) were relatively high and longer duration treatment with IFN-regimens led to higher costs with monitoring and medical visits (mean cost of PPP$ 6133.15 for IFN-based vs PPP$ 2830.42 for interferon-free regimens). In addition, adverse events are more frequent in IFN-based regimens than DAA treatment leading to higher costs. Previous studies reported that extra medical visits and drugs used for adverse events treatment, such as epoetin-α or filgrastim, lead to an increase in costs for treatment with IFN-based regimens with first generation PI (BOC/TEL) [36]. Costs per SVR might have 2-fold increase for patients with thrombocytopenia compared to those with normal platelet count when treated by IFN-based regimens [€50,907 (95% CI, 44,151–59,612) vs €26,105 (23,068–29,296)] [34].

Data from real-world cohort studies have been showing higher SVR rates by 12-week course of SOF/DCV (up to 92%) [17] and SOF/SIM regimens (up to 85%) [26] compared to IFN-based regimens (up to 40% in cirrhotic patients treated by PEG-IFN/RBV/BOC or TEL) [37]. However, presence of cirrhosis and GT subtype 1a might lead to lower SVR rates (Table 4). The extension of SOF/DCV for 24 weeks might be used to achieve higher SVR rates in cirrhotic patients [17]. In GT1 HCV infected patients, treatment using DAAs has been considered as cost-effective [38] and further analysis are needed to evaluate whether this approach is cost-saving [39]. In addition, treatment by SOF/DCV or SOF/SIM result in less medical visits and blood analysis tests for monitoring, lower rates of adverse events, no need of subcutaneous injection and lower intake of tablets/day compared to IFN-regimens. However, presence of decompensated cirrhosis highly increase the cost of treatment [from PPP$ 19,761.72to 36, 597.71 (from US$ 10,985.9 to 20,345.04] due to extension of SOF/DCV regimen for 24 weeks. Despite, the lack of cost-effectiveness studies in Brazil, given the fact that DAA-regimens are more effective compared to IFN-regimens and total cost for HCV treatment with all-oral interferon-free drugs is lower than IFN-based regimens, we could hypothesize that treatment with DAAs might be dominant in this country. Considering local costs and SVR rates reported in international real-life cohorts, the cost per SVR in Brazil could range from 20 to 30 thousand PPP$. Early viral kinetics might be used to individualize duration of DAA therapy decreasing costs by up to 20% [40]. On the other hand, treatment of patients with advanced fibrosis/compensated cirrhosis for 12 weeks, as recommended by the BMoH guideline, might lead to relative high rates of DAA failure or resistance and increased future costs for re-treatment. The addition of RBV (1250 mg PO daily) to SOF/SIM or SOF/DCV does not highly increase the cost of treatment due to local generic production and low price (US$ 706 or PPP$ 1273 per patient per 12 week duration). In Brazil, the prevalence of GT 1b seems to slightly higher than 1a (55% vs 45%) [41]. Considering our HCV prevalence and costs described in the present study, the financial burden could be up to PPP$ 21 billion (or US$ 12 billion) to treat all GT1 HCV infected people independently of fibrosis stage in Brazil. This amount represents approximately 13% of the general government expenditure on health in Brazil during 2014 [Health expenditure series. World Health Organization, available at: http://apps.who.int/nha/database/Select/Indicators/en; accessed at March 2017].

The main limitations of the present study are the lack of primary data on patient’s follow-up and costs of treatment for non-GT1 patients and new generation of interferon-free regimens. Presence of adverse events might lead to increased costs for medical care and low treatment adherence might result in higher costs and higher costs per SVR than our estimates, due to non-response/relapse. However, primary data on DAA-treatment remain scarce in Brazil because the strategy from BMoH for universal access to SOF, DCV and SIM has been recently implemented. We estimated the costs of an ideal follow-up for standard treatment of GT1, the most prevalent genotype, HCV patients according to recent BMoH’s recommendations. We are aware that treatment with fixed dose one-tablet daily of SOF/ledispavir (400/90 mg) or SOF/velpatasvir (400/100 mg) for 12 weeks has been recommended as first-line therapies for GT1 patients with or without cirrhosis [42]. These patients can also be treated with the fixed-dose combination of ombitasvir (12.5 mg), paritaprevir (75 mg) and ritonavir (50 mg) in one single tablet and dasabuvir (250 mg) [PTV/r/OMV + DSV regimen] or the fixed dose combination of grazoprevir (100 mg) and elbasvir (50 mg) in a single tablet once-daily [42]. However, these drug combinations are not currently available in Brazil for cost estimation. Another major limitation might be the fact that we evaluated the costs for HCV treatment in a short-term horizon and without prospective follow-up to evaluate whether DAAs treatment are cost-saving compared to PEG-IFN regimens.

The main strengths of this analysis are the PPP-adjustment for costs, the estimation of “real-price” for HCV-drugs and those related to monitoring/medical care accounting for the economic costs of public funds in medical investments. PPP adjustment is essential to allow comparison of prices of goods and services across countries. For high-income countries, PPP adjustment results in lower prices. In contrast, countries with weaker purchasing power, such as Brazil, have a significant increase in prices with PPP adjustment [43]. In the present study, the PPP-adjustment led to an increase in 1.8 fold in US$ costs. The cost for each drug were defined as the weighted-mean price of all HCV-drugs bought by the BMoH in 2015 and registered in web-based and open-access platform. In addition, we analysed micro-costs of all procedures recommended pre, during and post-HCV treatment to allow the estimation of medical care financial burden beyond drug costs.

## Conclusions

In conclusion, therapies with all-oral interferon-free, such as SOF/DCV and SOF/SIM for 12 weeks, have a high efficacy and need shorter treatment duration leading to lower costs for monitoring and medical care compared to IFN-based regimens in Brazil where DAAs had more than 90% discount over international prices. HCV treatment by IFN-free regimens will probably be associated with high financial impact worldwide. However, reduced DAA prices due to negotiation between health authorities and pharmaceutical companies can turn HCV treatment into an accessible strategy to HCV eradication.

## Additional file

Additional file 1: Micro-costing analysis of guideline-based treatment by direct-acting agents: the real-life case of hepatitis C management in Brazil. (DOCX 28 kb)

## Abbreviations

ABC: Activity based costing
ALT: Alanine aminotransferase
APRI: Aspartate-to-platelet ratio index
AST: Aspartate aminotransferase
BMoH: Brazilian Ministry of Health
BOC: Boceprevir
BRL: Brazilian Real
CHC: Chronic hepatitis C
DAA: Direct-acting antiviral drugs
DCV: Daclastavir
FIB-4: Fibrosis-4 score
GDP: Gross domestic product
GT1: Genotype 1
HCV: Hepatitis C virus
PEG-IFN: PEG-interferon
PPP$: Purchasing power parity Dollars
RBV: Ribavirin
SELIC: Special System for Settlement and Custody
SIM: Simeprevir
SOF: Sofosbuvir
SUS: *Sistema Único de Saúde*
SVR: Sustained virological response
TE: Transient elastography
TEL: Telaprevir
US$: United States Dollars
