# Supplementary material for: Micro-costing analysis of guideline-based treatment by direct-acting agents: the real-life case of hepatitis C management in Brazil
Source: BMC Gastroenterol. 2017 Nov 23;17:119. doi: 10.1186/s12876-017-0676-8 (PMC5701370; doi:10.1186/s12876-017-0676-8)
Supplement: Additional file 1: — Micro-costing analysis of guideline-based treatment by direct-acting agents: the real-life case of hepatitis C management in Brazil. (DOCX 28 kb) [file 12876_2017_676_MOESM1_ESM.docx]

**Additional file 1**

**Micro-costing analysis of guideline-based treatment by direct-acting agents: the real-life case of hepatitis C management in Brazil**

Hugo Perazzo, Marcelino Jose Jorge, Julio Castro Silva, Alexandre Monken Avellar, Patrícia Santos Silva, Carmen Romero, Valdilea Gonçalves Veloso, Ruben Mujica-Mota, Rob Anderson, Chris Hyde, Rodolfo Castro

| **Supplementary Text** | **Page** |
| --- | --- |
| **Cost of non-invasive methods for liver fibrosis staging** | 1 |
| **Supplementary Tables** |  |
| **Table S1.** Search strategy for identification of studies that evaluated efficacy of sofosbuvir/daclastavir and sofosbuvir/simeprevir with or without ribavirin for hepatitis C treatment in a real-life scenario | 2 |
| **Table S2.** Cost per unit of transient elastography by FibroScan® at INI/FIOCRUZ, Rio de Janeiro, Brazil | 3 |
| **Table S3**. Monitoring and drugs costs (US$ and PPP-adjusted US$) for treatment of genotype-1 patient with chronic hepatitis C and decompensated cirrhosis (Child-Pugh B/C) according to the BMoH from 2015 | 4 |
|  |  |

**Cost of non-invasive methods for liver fibrosis staging**

The costs of the following non-invasive methods for liver fibrosis staging were calculated: (i) aspartate-to-platelet ratio index (APRI), (ii) fibrosis-4 score (FIB-4) and transient elastography (TE). The price of the parameters used to calculate APRI [aspartate aminotransferase (AST) levels and platelet count] and FIB-4 [AST, alanine aminotransferase (ALT) levels and platelet count] were taken into account for estimating their costs. The following aspects were taken into account to estimate the cost per unit of TE (i) the price and depreciation of the FibroScan® machine; (ii) annual maintenance by M probe calibration; (iii) salaries of personnel who perform the examination; (iv) indirect administrative costs and (v) the opportunity cost of using public resources for implementation of new technologies (cost of capital). Personnel cost was defined as the proportional salary of an experimented operator according to time dedicated to TE exams. Indirect administrative costs (overheads) were based on proportion of operational costs, such as building, energy, water use and consumables, which were related to the activity of TE examination.

The cost per unit of TE examination was calculated considering the performance of 2,880 TE tests in 12 months (i.e., 15 exams per business day). Financial and economic perspectives for cost estimation were calculated for TE. Costs by the financial perspective were defined by the budget needed to have the service available. The economic perspective was defined as the costs calculated for the financial perspectives plus the opportunity cost of medical equipment. Considering the public health system perspective, the opportunity costs consist of the resources that could have been received in terms of remuneration of public bonds if the investment in the FibroScan® machine was not done. The Special System for Settlement and Custody (SELIC) is a rate that the Brazilian government uses to raise funds by selling public bonds in the market. As an example, the average SELIC’s rate in 2015 was 13.25% per year. The opportunity cost for TE was calculated considering the sum of the values ​​of all the other cost items related to the exam using the SELIC’ rate for an estimated 10-year useful machine’s life and the annual discount rate corresponding to the central inflation target regimen of the Central Bank of Brazil (4.5% per year).

**Table S1.** Search strategy for identification of studies that evaluated efficacy of sofosbuvir/daclastavir and sofosbuvir/simeprevir with or without ribavirin for hepatitis C treatment in a real-life scenario

| **#** | **Searches** | **Results** |
| --- | --- | --- |
| 1 | Sofosbuvir [All fields] | 1437 |
| 2 | Sovaldi [All fields] | 1439 |
| 3 | GS-7977 [All fields] | 1437 |
| 4 | Simeprevir [All fields] | 648 |
| 5 | Olysio [All fields] | 648 |
| 6 | TMC-435 [All fields] | 650 |
| 7 | Daclastavir [All fields] | 635 |
| 8 | Daklinza [All fields] | 12 |
| 9 | BMS-790052 [All fields] | 256 |
| 10 | Hepatitis C [All fields] | 80194 |
| 11 | Real-world [All fields] | 222815 |
| 12 | Real-life [All fields] | 11541 |
| 13 | #1 OR #2 OR #3 OR #4 OR #5 OR #6 OR #7 OR #8 OR #9 | 1859 |
| 14 | #11 OR #12 | 33927 |
| 15 | #10 AND #13 AND #14 | 150 |

Search carried out in PubMed on 06/08/2017

**Table S2.** Cost per unit of transient elastography by FibroScan® at INI/FIOCRUZ, Rio de Janeiro, Brazil.

|  | **Cost** | | **% of total** | |
| --- | --- | --- | --- | --- |
|  | **US$** | **PPP$** | **Without the opportunity cost** | **With the opportunity cost** |
| Personnel | 22.28 | 40.08 | 42.0 | 20.5 |
| Machine / depreciation | 16.57 | 29.80 | 31.3 | 15.3 |
| Maintenance of the machine | 0.37 | 0.66 | 0.7 | 0.3 |
| Indirect Administrative costs | 14.14 | 24.78 | 26.0 | 12.7 |
| Opportunity costs | 55.19 | 99.93 | - | 51.2 |
| **Total cost without the opportunity cost** | **53.35** | **95.32** | **100%** | **-** |
| **Total cost with the opportunity cost** | **108.54** | **195.25** | **-** | **100%** |

Costs were collected in Brazilian Real in 2013 prices and they were inflated to average prices of July of 2015 in United States Dollars (US$) and with purchasing power parity (PPP$).

**Table S3.** Monitoring and drugs costs (US$ and PPP-adjusted US$) for treatment of genotype-1 patient with chronic hepatitis C and decompensated cirrhosis (Child-Pugh B/C) according to the BMoH from 2015.

|  | **SOF/DCV 24w** | |
| --- | --- | --- |
|  | **US$** | **PPP$** |
| TE | 53.35 | 95.32 |
| **Total** | **53.35** | **95.32** |
| **Blood analysis tests** |  |  |
| WBC. RBC and platelet | 63.76 | 114.69 |
| Fasting glucose | 29.2 | 52.52 |
| BUN / creatinine | 29.03 | 52.22 |
| ALT | 29.11 | 52.37 |
| AST | 29.17 | 52.47 |
| AP | 28.61 | 51.46 |
| GGT | 28.99 | 52.15 |
| Total bilirubin | 29.04 | 52.24 |
| Albumin | 30.44 | 54.75 |
| INR | 40.1 | 72.14 |
| HCV genotype | 156.33 | 281.22 |
| HCV-RNA | 174.11 | 313.2 |
| TSH |  | - |
| Beta-hCG | 182.07 | 327.53 |
| **Total** | **849.96** | **1528.96** |
| **Medical follow-up** |  |  |
| GP's medical visit | 54.54 | 98.11 |
| Specialist's visit | 314.62 | 565.97 |
| Blood sample | 227.01 | 408.36 |
| Nursing consultation | 127.32 | 229.03 |
| **Total** | **723.49** | **1301.47** |
| **Drugs** |  |  |
| PEG-IFN 180 mcg | - | - |
| Ribavirin 250 mg | - | - |
| Boceprevir 200 mg | - | - |
| Telaprevir 375 mg | - | - |
| Sofosbuvir 400 mg | 13531.36 | 24341.36 |
| Daclastavir 60 mg | 5186.88 | 9330.60 |
| Simeprevir 150 mg | - | - |
| **Total** | **18718.24** | **33671.96** |
| **TOTAL COST** | **20345.04** | **36597.71** |

Costs in United States Dollars (US$) and with purchasing power parity (PPP$) on July 2015. Drug costs estimated as the weighted-mean price (http://www.saude.gov.br/bps). PEG-IFN, peginterferon; WBC, White blood cell; RBC, red blood cell; BUN, blood urea nitrogen; ALT, alanine aminotransferase; AST, aspartate aminotransferase; AP, alkaline phosphatases; GGT, gamma-glutamyl transpeptidase, INR, international normalized ratio; TSH, thyroid-stimulating hormone; hCG, human chorionic gonadotropin; GP, general practitioner
